# Testing the Feasibility and Potential Impact of a Mindfulness-Based Pilot Program in Urban School Youth

**DOI:** 10.3390/ijerph19063464

**Published:** 2022-03-15

**Authors:** Sabrina Krebs, Emily Moak, Shakiba Muhammadi, David Forbes, Ming-Chin Yeh, May May Leung

**Affiliations:** 1 Nutrition Program, Hunter College, City University of New York, 2180 Third Avenue, New York, NY 10035, USA; sabrina.krebs77@myhunter.cuny.edu (S.K.); myeh@hunter.cuny.edu (M.-C.Y.); 2 School of Public Health, City University of New York, 2180 Third Avenue, New York, NY 10035, USA; emily.moak@colorado.edu (E.M.); shakiba.muhammadi@mountsinai.org (S.M.); 3 Urban Education Doctoral Program, Graduate Center, City University of New York, 365 Fifth Avenue, New York, NY 10016, USA; dforbes@brooklyn.cuny.edu

**Keywords:** mindfulness, mindful eating, childhood obesity

## Abstract

Mindfulness-based interventions (MBIs) could be effective in engaging children and reducing childhood obesity risk. The purpose of this study was to test feasibility, fidelity, and potential impact of a pilot MBI in urban school youth. A two-group quasi-experimental study was conducted in a Harlem, New York school. Participants comprised 51 students (ages 9–12, 54% female, 85% African American/Black). The experimental (E) group (*n* = 26) participated in a nine-session pilot MBI. Sessions were 90 min and offered weekly as part of afterschool programming. Children only attending during the school day comprised the control (C) group (*n* = 25). Process evaluation (e.g., fidelity, reach) was performed. Interviews with the E group were conducted to determine program acceptability. Mindful eating and resilience measures were collected at baseline and post-intervention. Intervention feasibility was high as the retention rate was 100% and fidelity was good as nine out of ten sessions were implemented. Relative to baseline, significant improvements were observed in the C group compared to the E group in the resilience composite score (*p* = 0.01) and its confidence domain (*p* = 0.01). A MBI may provide a unique opportunity to engage youth. However, further research is warranted to determine if a MBI could promote health in urban, school-age children.

## 1. Introduction

Childhood obesity (CO) continues to be a serious clinical and public health challenge in the United States (USA). Over the last three decades, the rate of CO has tripled, with more than one-third of children ages 6 to 19 now considered overweight or obese [1,2]. In New York City (NYC) alone, nearly 40% of public-school children in grades K-8 are overweight or obese, with low-income neighborhoods reporting higher rates than the rest of NYC [3]. CO has profound short- and long-term consequences: it increases the risk of metabolic disturbances, such as dyslipidemia and insulin resistance, compromises a child’s quality of life, and increases the risk of adult morbidity and mortality [4,5,6,7,8].

CO also leads to extensive economic costs; it is estimated that CO raises annual medical care costs by $907 or 92% per child [9]. In the USA, CO is estimated to cost up to $14 billion dollars in direct medical costs annually [10]. Obesity affects children disproportionately from low-income families, especially Black youth. The prevalence of CO is reported to be higher in non-Hispanic Blacks (24.2%) than among non-Hispanic Whites (16.1%) [11].

Furthermore, youth residing in low-income urban neighborhoods are at greater risk of obesity and have elevated environmental stressors compared to more economically advantaged peers [12]. High rates of crime and violence in these neighborhoods often prevent youth from engaging in physical activity (PA) outdoors [13]. Hence, these youth tend to adopt more sedentary behaviors due to the limitations of urban indoor environments, further increasing their risk of being overweight and obese [14]. It is important to develop effective and innovative interventions to address CO in populations at greatest risk, while also considering the environmental context and resources.

### Theoretical Framework: Why Mindfulness Interventions May Be an Effective Way to Promote Positive Behaviors to Reduce the Risk of CO

Mindfulness interventions focused on nutrition were initially developed to treat eating-disordered adult patients, and such interventions have been shown to be effective in reducing weight-related outcomes and associated disease risks through improved dietary behaviors in adults [15,16,17,18,19]. Thus far, the majority of mindfulness-based interventions (MBIs) have targeted older individuals/adults, while few interventions have been conducted in the youth population, primarily aimed at reducing mental stressors, such as anxiety and depression and improving self-esteem [20,21,22,23,24,25]. Mindfulness theoretically may improve eating behaviors through promoting self-regulation, as these interventions are designed to foster awareness and attention to thoughts, emotions, and actions related to lifestyle behaviors critical for health [26]. MBIs may appeal to youth because the self-management practices taught can enable them to have a significant role in their own growth and development [27]. Studies have also shown youth’s receptivity to MBIs due to their openness and readiness for new ideas and experiences [26,27].

Some researchers have begun to explore the impacts these interventions have on CO and related co-morbidities. One study, which focused on promoting PA with a 12-week Ashtanga yoga intervention among Hispanic youth at risk for type 2 diabetes, reported a significant average weight loss of 2 kg [28]. Anxiety symptoms also reduced in the participants. A 6-week mindful eating intervention conducted among obese Latino adolescents resulted in a significant decrease in body mass index (BMI) by 1.1 kg/m^2^ in the intervention group, and after the intervention ended, BMI continued to decline to 1.4 kg/m^2^ at week 10 [29]. Other MBIs have resulted in improved eating behaviors and attitudes among adolescent girls at a heightened risk for type 2 diabetes [30,31]. Another mindfulness intervention study in youth with type 1 diabetes saw reductions in overeating [32].

Social cognitive theory (SCT), which has informed the design of effective behavior change interventions for youth, offers explanation to ways in which a MBI may influence health and, more specifically, CO-related behaviors [33]. Observational learning is a common construct used extensively to promote positive changes. By observing a role model, individuals can learn a behavior and be more likely to perform it, if they see the model rewarded for the behaviors in ways they value [33]. The efficacy of SCT constructs in predicting CO-prevention behaviors has previously been assessed among upper elementary African American children, which revealed that self-efficacy was a significant predictor for eating fruits and vegetables, and self-efficacy and self-control were significant predictors for drinking water [34]. Another SCT-guided CO-prevention intervention showed that overweight 5- to 10-year-olds significantly improved their dietary self-efficacy compared with their normal-weight and at risk of overweight counterparts [35]. Inner-city African American school children benefited from varying sources of social support and moderate levels of self-efficacy in increasing PA levels [36]. Additionally, though targeting rural families, a study on mindful eating showed that role modeling significantly improved mindful eating behaviors [37].

This theoretical framework, along with the promising findings from prior studies, highlight the potential impact of MBIs as an approach to address CO. Thus, a comprehensive MBI incorporating mindful eating, yoga, and stress reduction could provide a unique opportunity to not only engage an often hard-to-reach minority youth population, but also reduce the risk of CO and promote mental well-being, while living with several environmental stressors.

The purpose of this study was to test the feasibility, fidelity, and potential impact of a 10-week mindfulness-based pilot intervention on mindful eating and resilience in low-income, urban middle-school youth.

## 2. Materials and Methods

### 2.1. Design and Procedures

We utilized a two-group quasi-experimental design with repeated measures before and immediately after the intervention period. The control (C) group included 4th and 5th grade students who did not attend afterschool programming, while the experimental (E) group comprised 4th and 5th grade students who did attend afterschool programming. Afterschool programming was a service provided by the school for working families, wherein children could engage in supervised activities (e.g., fitness classes, homework support, arts and crafts projects) after school until 6 pm each weekday. A school administrator identified a 4th and 5th grade class comprising students who did not attend afterschool programming to be the control group.

Participant assent and parental permission were first obtained. All participants were informed that they would receive a $20 gift card upon completion of the study. Those who participated in the intervention also received a yoga mat at the end of the study. Prior to the start of the intervention, the program was introduced, and pre-intervention questionnaires were conducted in a classroom setting. Following this, the E group met weekly; the C group did not meet during the intervention period. Post-intervention measures were repeated for both groups at the end of the intervention. Interviews were also conducted post-intervention with the E group and a convenience subsample of their parents by trained RAs. The study was approved by the City University of New York (CUNY) Institutional Review Board.

### 2.2. Setting and Participants

This feasibility and pilot study was conducted during Spring 2014 in an afterschool program at an urban private Evangelical school in Harlem, New York, where the majority of students were Black. Inclusion criteria included youth who were in grades 4 and 5 (mean age 10.0 ± 0.7 years) and wrote and spoke English. Other criteria for the E group included participation in afterschool programming, physical ability, and availability to meet weekly for 10 sessions. The intervention was held once per week immediately after school in a classroom or gym setting.

### 2.3. Intervention

The intervention was guided by SCT. A summary of how specific SCT constructs were operationalized in our intervention is presented in Table 1. Briefly, the instructor was of the same racial/ethnic background as the participants, with the goal of participants responding more positively to the instructor’s messages. The instructor also demonstrated the yoga postures and participated in the yoga and mindful eating activities. This use of a role model to perform new behaviors for the students aimed to more effectively improve outcome expectations, behavioral capability, and self-efficacy [33]. Discussions with the instructor and handouts promoting yoga and healthy eating were also incorporated to provide opportunities for didactic learning and reinforcement of messages related to healthy dietary and PA behaviors. The intervention was also adapted from the “Learning to BREATHE” mindfulness curriculum by Patricia Broderick [38].

The intervention sessions were led by a certified yoga instructor who has taught mindfulness in youth and is a former schoolteacher who instructed in underserved communities. The intervention consisted of ten 90 min sessions that were held weekly. The intervention had three components, which included PA in the form of yoga, nutrition in the form of mindful eating, and overall mindfulness in the form of mindful breathing. Of the allotted 90 min, 20 min were for yoga, 20 min for a mindful eating activity, and 45 min for mindfulness/mindful breathing. The yoga component consisted of a slow flow, mini vinyasa practice with the goal of building a yoga pose vocabulary, practicing mindful breathing, and noticing sensations in the body. Students were encouraged to pay attention to their thoughts and emotions. The mindful eating component consisted of discussion and activities addressing three key domains of mindful eating: awareness, external cues, and distraction. The mindfulness component consisted of breathing techniques and guided mindfulness practice, which included such activities as a body scan, where each student was encouraged to pay attention to how different areas of his/her body felt in the present moment. Students were encouraged to recall senses when eating mindfully, apply mindfulness practices to respond rather than react to situations, and discuss feelings in order to reduce stress and calm the mind. A home challenge was also presented at the end of each session to encourage the practice and application of knowledge and skills learned in real-world settings. A summary of the curriculum can be seen in Table 2.

### 2.4. Measures

#### 2.4.1. Feasibility, Fidelity, Acceptability, and Perceived Impact of Intervention

The benchmark for feasibility was an average of 55% of the participants would attend each of the 10 sessions, as informed by a previous mindful eating intervention conducted with minority youth [29]. Participant attendance was taken at the beginning of each session by a trained research assistant (RA). Another measure of feasibility included engagement, which was defined as the extent to which the youth paid attention to the instructor and were focused on the curriculum tasks. To assess engagement, RAs, who provided classroom management support, observed participants’ level of focus vs. distraction (e.g., side conversations, needing reminders to pay attention). One RA was assigned to rate engagement for the three components of each session, on a 4-point scale ranging from *None of the time (1)* to *All of the time (4)*.

Fidelity, defined as the extent to which the intervention was implemented as intended, was also assessed by the number of sessions and activities within each session that were actually implemented, and distribution of materials, which were collected by a trained RA. Intervention activities were determined as *very successful* if they were implemented as originally designed. They were determined as *mostly* or *partly successful* if parts were not implemented fully or adjustments were needed to be made to support the implementation process.

Acceptability and satisfaction were assessed by interviews with the intervention participants and their parents. Specifically, in the interviews with participants, questions were asked about perceived impact, knowledge learned during the intervention, and if the youth had adopted any relevant behavior changes. Sample questions included “What are some benefits of mindfulness?” and “What are some situations that might be helpful to use mindfulness?” Questions related to whether children discussed the intervention and/or practiced mindfulness at home were asked in the parental interviews. Sample questions included “Have you noticed any changes in your child over the last few weeks?” and “Has your child mentioned anything about the study or its activities with you?”

#### 2.4.2. Psycho-Social Outcomes

Both E and C group participants completed psychosocial measures at baseline and immediately after the last intervention session.

The Mindful Eating Questionnaire (MEQ) (Framson et al., 2009) contains five domains: disinhibition (inability to stop eating even when full), awareness (being aware of and appreciating the effects of food on the senses), external cues (eating in response to environmental cues), emotional response (eating in response to negative emotional states), and distraction (focusing on other activities while eating) [39]. While originally designed and validated in an adult population, 14 of the original 28 items were adapted for use with the current adolescent study population. Questions were adapted by removing the disinhibition and emotional response domains entirely, which had lower internal correlations when tested among children [40]. This decision was informed by conversations we had with school instructors to review the initial questionnaires for age appropriateness and length. The instructors believed that it was too complex for youth to respond accurately to concepts addressed in these domains. Several items were also reworded to improve comprehension by the study population, based upon feedback from school instructors. Examples of changes included modifying the statements “I notice when there are subtle flavors in the foods I eat” to “I notice when there are slightly different flavors in the foods I eat” and “I notice when the food I eat affects my emotional state” to “I notice when the food I eat makes me feel happy or sad.”

The *Adolescent Resilience Questionnaire (ARQ)* (Gartland et al., 2011) comprises 12 scales within five domains: individual, family, peer, school, and community. While originally designed and tested for validity with adolescents living with a chronic illness, this questionnaire was adapted for the current study population in a similar process as the MEQ was [41]. For the purpose of the present study, 26 items were included from the original 93-item questionnaire. Only items from the individual domain were used, with questions from the following scales: confidence, emotional insight, negative connotation, social skills, and empathy. Four items were deleted from the original ARQ because the curriculum does not discuss social support. One item was deleted because of similarity to another question, and two items were deleted because the concepts were considered beyond our population’s understanding. For example, item 5 (“My life has a sense of purpose”) contained a concept considered beyond our population’s understanding. There were also concerns about the negative items on this scale (e.g., negative cognition, some items related to social skills) for the target population. With the goal of shortening the survey to about 20 questions (to minimize participant burden) the negative cognition domain and some of the social skills questions about helplessness were removed. Neither the modified MEQ nor the modified ARQ was tested for validity and reliability.

All items for both surveys were measured on a 5-point scale ranging from *Almost Never (1)* to *Almost Always (5)*.

### 2.5. Statistical Methods

Quantitative data from surveys were analyzed using Statistical Package for the Social Sciences (SPSS) Software (Windows Version 28; IBM, Chicago, IL, USA). Demographic data were analyzed with chi-squared tests. Mann–Whitney U tests were performed to detect differences in the change of scores in overall domains for the psycho-social measures, from baseline to post-intervention between groups. Significance was defined as a *p*-value < 0.05.

Interviews were audio taped and transcribed verbatim. A preliminary codebook was developed, guided by an initial review of randomly selected transcripts. The primary researcher and an RA coded the transcripts to identify codes (ideas emerging from text). Reflexive iteration was used to identify additional codes. The two researchers compared coding to ensure consistency and resolved any discrepancies through discussion and consensus. Data reduction then occurred where similar codes were grouped together to identify relevant themes. Data analysis was conducted using thematic conceptual matrix sheets with Dedoose (version 5.2; Scientific Software, Hermosa Beach, CA, USA).

## 3. Results

### 3.1. Participant Characteristics

A total of 51 participants were allocated to either the E (*n* = 26) or the C (*n* = 25) group, as seen in Table 3. The average age of the E group was 10.575 years (SD = 0.622) and of the C group it was 10.455 years (SD = 0.800). At baseline, the E group consisted of 13 females (52.0%) and 12 males (48.0%), while the C group consisted of 14 females (56.0%) and 11 males (44.0%). Participants in both groups were predominantly Black (E: 88.0% vs. C: 84.0%). Six participants (30.0%) from the E group and five participants (23.8%) from the C group reported consuming fruits and vegetables five or more times per day in the past seven days, respectively. Ten participants (40.0%) from the E group and six participants (24.0%) from the C group reported participating in 60 min of activity daily the past seven days, respectively. No significant differences were found at baseline between the E and C groups for the demographic variables of sex, race, ethnicity, fruit/vegetable intake, or PA behavior.

### 3.2. Feasibility

Of the 26 students in the E group, 25 (96.2%) completed the survey measure at both time points. Attendance of the 26 students in the E group averaged 73.5% across the sessions (M = 19.11, SD = 5.06). Sessions 4 and 7 had the fewest students attending, which was 12 students (46%), while the first and final sessions had the highest attendance with 25 and 26 students (96% and 100%), respectively. Thus, by the final session, all 26 of the original participants remained in the intervention (100% retention rate).

Youth participation was also measured by evaluating their engagement during each of the sessions. For 55.6% of the sessions, students were engaged *most* to *all of the time*, while another 20.1% of sessions, students were engaged *part of the time*.

#### Fidelity

Overall, intervention fidelity was good. Of the 10 planned sessions, nine of them were conducted. One session (session 9) was canceled due to a conflict with a planned school event. Specifically, of the nine sessions conducted, 55.6% of the yoga and mindful eating sessions, and 66.7% of the mindfulness activities were *very to mostly successful* in their implementation. Handouts were given to the students in eight of the nine sessions (88.9%).

### 3.3. Acceptability

Throughout the interviews, E group participants mentioned several benefits that were either related to concepts taught during the program or to the use of new skills that were gained through the intervention. In regard to concepts learned, participants specifically articulated their perception of the benefits of mindfulness and mindful eating. One student mentioned that *“when I was breathing my stress came out”*.

The ability to apply mindful eating in their daily lives was also mentioned by most participants as a benefit of the program. The children specifically described different situations in which they applied mindful eating, showing comfortable familiarity with this concept and a clear idea of its benefits. Several participants identified practicing mindful eating with eating at a slower pace, and consequently, savoring their meals. Similarly, many of the participants reported that because of the mindfulness eating techniques taught in the program, they paid more attention to the food they are eating in their day-to-day lives. This includes noting the smell, texture, shape, and taste of food. One child noted that *“every time I eat food...I feel it”*. Some participants also reported that they made better and healthier food choices.

Another benefit noted by many of the participants was the opportunity to practice yoga as a form of mindfulness and PA. Engagement in this intervention study was the first time many of the students had ever practiced yoga. Many expressed that they had favorite positions they found to be challenging but also stimulating and calming. A few also articulated that they enjoyed learning new poses and positions and demonstrated them to their siblings and parents. One student shared that *“I felt my muscles getting stronger”* (in response to a question about the effects/benefits of doing the yoga).

Other specific benefits of the intervention identified by the participants included improved interpersonal skills, managing stressful situations, and school performance improvement. As one student said, *“when I do something bad at school and I come home from school, and I get in trouble I go to my room and instead of punching something I take a deep breath”*. Participants also provided several examples of how breathing and thinking before reacting helped them in stressful situations.

A convenience sub-sample of parents (*n* = 6) were interviewed as well to understand perceived benefits. They shared that their children enjoyed the yoga activities and demonstrated the yoga movements they learned at home. Parents reported that their children were eating healthier but had not heard anything about the mindfulness component. Parents were unfamiliar with the home challenges that the students were instructed on. One parent mentioned her daughter requested to be picked up later because she was enjoying the program, and particularly liked the yoga component.

### 3.4. Psychosocial Domains

Relative to baseline, significant improvements were observed in the C group compared to the E group in the ARQ confidence domain (*p* = 0.01) and the ARQ composite score (*p*-value = 0.01), as seen in Table 4. No significant differences were observed between E and C groups for improvements in the ARQ emotion, negative, social and empathy domains or any of the MEQ domains and composite scores.

## 4. Discussion

The purpose of this study was to test the feasibility, fidelity, and potential impact of a mindfulness-based pilot intervention on psycho-social variables related to mindful eating and resilience in urban middle-school youth.

A retention rate of 100% in the current study is higher than previous studies [29]. Of the 26 students in the E group, 25 (96.2%) completed the survey measures at both time points. Despite challenges with conducting the intervention at the end of the school week, the students showed willingness as reflected not only in the high retention rate but also in the average total participation rate of 73.5% across the sessions. This program also achieved high fidelity with 90% of intended sessions conducted and handouts given in 88.9% of sessions, demonstrating that such an intervention can be implemented with limited resources.

The participants evaluation of the intervention was positive, including gaining new skills, ability to apply techniques to their daily lives, improved management of stress, and improvement in school performance. The feasibility and acceptability findings support, to some extent, what has been reported in previous studies, highlighting that this intervention could be an engaging approach with this at-risk population [20,25,29,31,44]. For example, the parental evaluation of the 6-month Foodie-U mindfulness curriculum indicated that lessons and activities were easy to follow and well-liked, and all 10 teachers involved agreed that the program’s lessons were effective [44]. In a study of a satiety-focused mindful eating intervention among adolescent Latina females, there was a retention rate of 57% in the E group and 65% of the C group [29]. A mindfulness-intervention study for urban youth showed 73.5% of students at one intervention school and under 50% of students at the second intervention school completed at least 75% of the intervention classes [20]. Three focus groups among students indicated that students generally had a positive experience in the program, and one focus group among teachers indicated uniform acceptability of training urban youth using yoga and mindfulness-based techniques.

In our study, the C group showed a significant improvement in confidence (ARQ domain) and overall resilience (ARQ composite score) compared to the E group. However, it is worth noting that at post-intervention both the confidence and overall resilient scores were comparable between the groups. Meanwhile, no other significant differences were observed between E and C groups.

Limited statistically significant changes from pre- to post-intervention in the experimental group compared to the control were found in studies with similar mindfulness interventions [22,28,31]. In Khalsa et al., statistically significant differences between groups were found for only a few outcome measures [22]. Benavides and Caballero noted that in their yoga intervention, even though improvements in self-concept, anxiety, and/or depression were seen in six participants, three participants had worsening self-concept and/or depression measures [28]. In Shomaker et al., there were no significant differences in perceived stress, and no significant between-condition effects on parent-reported or behavioral measures of executive function [31]. However, researchers noted that despite these study limitations, exploratory and qualitative findings highlighting mental well-being benefits among some participants demonstrate that MBIs are still a worthwhile approach that warrant further exploration [22,28,31].

The current study’s quantitative findings were inconsistent with its qualitative findings, possibly due to several limitations related to study design and implementation. The ARQ measurement tool was originally validated for adolescents, whereas the current study’s participants were younger (in grades 4 and 5). Further, the modified ARQ tool used in our study was not tested for validity or reliability. The setting for the completion of the post-intervention questionnaire differed between the E and C groups. The E group completed their questionnaire during the final session, where there was a program-end celebration in a gym setting, while the C group completed their questionnaire in a quiet classroom setting. Additionally, due to feasibility issues, a quasi-experimental study design was implemented with the C group comprising students who did not attend any afterschool programming. Differences in potential confounders, such as poverty status, engagement level, and food security, have been found among children who participate in afterschool activities versus children who do not [45]. These potential socio-demographic differences between the two groups could have impacted the study measures at both baseline and post-intervention.

While intervention fidelity was good, implementation of the session components could have been improved upon as 56 to 67% of the activities were considered *very* to *mostly successful*. Possible reasons for these implementation challenges included the timing of the intervention, which occurred at the end of the school day and school week (Friday afternoons), so students were more easily distracted, some of the sessions were held in smaller classrooms with classroom materials in reach, and for one of the sessions, students had recently returned from their spring break holiday, resulting in more side conversations. Potential approaches to address these challenges in future studies and interventions, if resources allow, include reducing the intervention group size so participants receive more individual attention and instruction; reduce the length of intervention sessions while increasing frequency of these sessions to maintain engagement and attention of participants; and ensure assistants are present to provide classroom management support. Furthermore, as per the convenience sub-sample of parents who reported that they were unfamiliar with the weekly home challenges which had been given to their children, the program could be adapted to incorporate parental involvement, which may lead to greater reinforced positive messaging in the home environment.

In addition to the limitations already discussed, this study included a small, non-powered sample size that was largely limited due to the size of the afterschool program. Data saturation also was not achieved when interviewing the parents. Furthermore, generalizability of the study findings is limited given that the sample comprised urban, predominantly Black school children attending afterschool programming.

Future research should include randomized controlled trials that minimize potential confounders, which also incorporate valid and reliable quantitative measures specifically designed for the intended population. This could provide further understanding about how a mindfulness-based approach focused on healthy eating, physical activity, and overall well-being may reduce the risk of childhood obesity among diverse, minority children.

## 5. Conclusions

In summary, the present study aimed to test feasibility and potential impact of a MBI on mindful eating and resilience. Quantitative data did not show any positive change in mindful eating or resilience measures in the E group, possibly due to the potential limitations noted above. However, the high feasibility and acceptability of this pilot study by the participants and parents and participant-reported application of skills that were learned during the intervention should be taken into consideration. Future research should be explored to gain a better understanding of the potential impact of this MBI and ultimately provide more insight into the potential impact of this relatively novel approach to engage diverse, minority children at risk of childhood obesity.

## Figures and Tables

**Table 1:** Summary of the Applications of Social Cognitive Theory in the Intervention.

Construct	How it was Applied
Behavioral capability	Activities, discussions, and handouts provided didactic learning and opportunities to practice skills
Outcome expectations	Activities and discussions highlighted the physical, mental and social health benefits of mindfulness
Self-efficacy	Curriculum was designed to make small, incremental changes; skills were built upon each other to evolve into more complex skills
Observational learning (modeling)	Instructor (of similar racial/ethnic background as participants), demonstrated role-modeled behavior and skills
Reinforcement	Home challenges included to create opportunities to practice and apply newly learned skills

**Table 2:** Summary of Planned Intervention Curriculum.

Session	Mindful Eating	Yoga	Mindfulness	Home Challenge
1	Crunch contest	Back to Mountain Yoga	Mindfulness of Sound; Mindful breathing	Mindful Breathing
2	Raisin exercise	Basic yoga poses	Mindfulness of Sound; My Mindful/Mindless Life; Body Scan	Mindful Breathing
3	Big vs. small bites	Yoga string spider web	The Big Event activity	Mindful eating at home
4	Learning to eat mindfully	Mindful positions & chime activity	My mind is a cast of characters activity	Mindful eating at home
5	Pairing foods to change flavor	Name the Yoga Pose activity	Mindfulness-of-feelings	Practice mindfulness of feelings activity
6	Triggers in my eating environment	Rock, Tree, Bridge activity	How does it feel? & Surfing the waves activities	Practice mindfulness of feelings activity
7	Hunger cues, sensory triggers, and awareness	Tall tree, small tree activity	“A Stressed-Out Case” Cross the line activity	Mindful yoga/stretching
8	Identify two mindful eating skills that I can apply to my everyday life	When the Big Wind Blows activity	Healthy mind habits & Practicing Meanness activities	Do something nice for yourself
9	Raisin activity	Yoga, Yoga, Pose! activity	Short mindfulness practice	Mindful yoga/stretching
10	Mindful meal celebration	Yoga freeze dance, hula hoop race	Designed to “re-mind” art project activity	Youths were given an anchor charm as a reminder to stay “grounded”, a yoga mat to continue practicing yoga, and a postcard with mindfulness messages.

**Table 3:** Summary of Participant Characteristics.

		Experimental (%)	Control (%)	X^2^	*p*-Value
Sex	Male	48.0	44.0	0.081	NS
	Female	52.0	56.0		
Age (years)	9	12.5	28.0	8.876	NS
	10	79.2	40.0		
	11	4.2	28.0		
	12	4.2	4.0		
Race	Black	88.0	84.0	2.023	NS
	Native Hawaiian or Other Pacific Islander	4.0	0.0		
	Mixed Race	4.0	4.0		
	No response	4.0	12.0		
Ethnicity	Hispanic or Latino	8.0	16.0	0.762	NS
	Not Hispanic or Latino	88.0	80.0		
	No response	4.0	4.0		
Family members at participant’s home	Mom or Dad	80.0	68.0	3.061	NS
	Aunt or Uncle	0.0	4.0		
	Grandma or Grandpa	0.0	0.0		
	Multiple Family Members	16.0	28.0		
	No response	4.0	0.0		
Healthy consumption of fruits and vegetables ^a^	5 or more times per day in the past 7 days	30.0	23.8	0.200	NS
	Less than 5 times per day in past 7 days	70.0	76.2		
Healthy physical activity levels ^b^	60 min/day, 7 days/week or more	40.0	24.0	1.471	NS
	Less than 60 min/day, 7 days/week	60.0	76.0		

^a^ Healthy consumption of fruits and vegetables is defined as five or more times per day in the past seven days, as per the United States Department of Agriculture (USDA)’s definition [42]. ^b^ Healthy physical activity levels for children is defined as 60 min per day, seven days a week or more as per the Centers for Disease Control (CDC)’s recommendation [43].

**Table 4:** Mean (SD) Baseline and post-intervention values of psychosocial measures in the E and C groups.

	Experimental		Control		
Scale Domain	Baseline	Post	Baseline	Post	*p*-Value *
**MEQ**					
Awareness	21.44 (5.18)	21.67 (4.73)	19.00 (6.09)	19.95 (5.88)	NS
External	17.84 (7.31)	19.56 (6.89)	15.96 (4.77)	17.85 (4.04)	NS
Distraction	10.28 (4.04)	10.44 (2.10)	10.80 (3.27)	11.05(3.25)	NS
Composite Score	49.56 (11.37)	51.67 (6.37)	45.76 (8.63)	48.85 (6.39)	NS
**ARQ**					
Confidence	28.40 (5.66)	28.41 (3.11)	23.04 (7.15)	28.30 (3.76)	0.01
Emotion	20.04 (5.19)	19.04 (6.17)	17.16 (5.15)	19.35 (5.83)	NS
Negative	13.92 (4.08)	18.93 (5.14)	15.80 (5.58)	20.65 (4.49)	NS
Social	9.36 (3.94)	9.54 (3.61)	7.12 (2.62)	9.45 (2.80)	NS
Empathy	12.28 (3.57)	12.46 (3.11)	12.04 (4.07)	12.90 (3.32)	NS
Composite Score	84.00 (13.18)	88.79 (15.49)	75.16 (14.19)	90.65 (13.67)	0.01

* *p*-value for Mann–Whitney U tests comparing improvements (baseline to post-intervention) between groups (E vs. C). There were no significant differences between groups, at baseline. MEQ = Mindful Eating Questionnaire; ARQ = Adolescent Resilience Questionnaire.

## Data Availability

The data that support the findings of this study are available from the corresponding author (M.M.L.), upon reasonable request.
